# [Retracted] Anthelmintic drug albendazole arrests human gastric cancer cells at the mitotic phase and induces apoptosis

**DOI:** 10.3892/etm.2024.12513

**Published:** 2024-03-26

**Authors:** Xuan Zhang, Jing Zhao, Xiangyang Gao, Dongsheng Pei, Chao Gao

Exp Ther Med 13:595–603, 2017; DOI: 10.3892/etm.2016.3992

Following the publication of the above paper, a concerned reader drew to the Editor’s attention that unexpected areas of similarity were noted in terms of the distribution of certain of the data featured for the ‘Control’ and the ‘0.1 μM ABZ’ panels in the flow cytometric experiments shown in Fig. 3B on p. 599, which were difficult to attribute to coincidence.

Following an internal enquiry, the Editor of *Experimental and Therapeutic Medicine* has been able to verify the claims made by the interested reader; therefore, in view of the abovementioned anomalies that were identified and owing to an overall lack of confidence in the presented data, the Editor has decided that the paper should be retracted from the Journal. The authors were contacted regarding these matters, and accepted the decision to retract this paper, with the exception of D.P., who proved not to be contactable. The Editor apologizes to the readership of the Journal for any inconvenience caused.

